# A New, Rapid Method for the Quantification of Dolichyl Phosphates in Cell Cultures Using TMSD Methylation Combined with LC–MS Analysis

**DOI:** 10.21769/BioProtoc.4880

**Published:** 2023-11-20

**Authors:** Dipali Kale, Timo Sachsenheimer, Albert Sickmann, Britta Brügger

**Affiliations:** 1 Heidelberg University Biochemistry Center (BZH), Heidelberg, Germany; 2 Leibniz-Institut für Analytische Wissenschaften-ISAS-e.V., Dortmund, Germany

**Keywords:** LC–HRMS, LC-MS, DolP, Dolichyl phosphate, TMSD, Methylation, Lipid analysis

## Abstract

Dolichyl phosphates (DolP) are ubiquitous lipids that are present in almost all eukaryotic membranes. They play a key role in several protein glycosylation pathways and the formation of glycosylphosphatidylinositol anchors. These lipids constitute only ~0.1% of total phospholipids, and their analysis by reverse phase (RP) liquid chromatography–high-resolution mass spectrometry (LC–HRMS) is challenging due to their high lipophilicity (log P > 20), poor ionization efficiency, and relatively low abundance. To overcome these challenges, we have introduced a new approach for DolP analysis by combining trimethylsilyldiazomethane (TMSD)-based phosphate methylation and HRMS analysis. The analytical method was validated for its reproducibility, sensitivity, and accuracy. The established workflow was successfully applied for the simultaneous characterization and quantification of DolP species with different isoprene units in lipid extracts of HeLa and *Saccharomyces cerevisiae* cells.

## Background

Dolichyl phosphates (DolP) are long-chain polyisoprenoid phosphates that function as glycan carriers in cellular N- and O-glycosylation, C- and O-mannosylation, and glycosylphosphatidylinositol-anchor biosynthesis (Orlean, 1990; Rosenwald et al., 1990). Numerous studies have reported altered endogenous DolP levels in pathophysiological conditions such as Alzheimer’s disease, dementia, and Prion disease (Kranz et al., 2007; Park et al., 2014; Sabry et al., 2016; Jaeken et al., 2020). Given the essential role of DolP in cellular glycosylation, there is a need for a rapid method to accurately characterize and measure DolP in biological samples, including those from patients with disorders associated with glycosylation defects.

**Figure 1.**
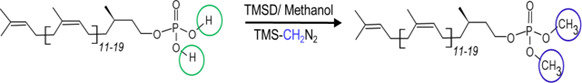
Chemical reaction of trimethylsilyldiazomethane (TMSD)-based methylation of dolichyl phosphates (DolPs). The simplified linear structure of DolP is displayed.

Generally, DolP are quantified after their fluorescence derivatization by combining normal-phase liquid chromatography (LC) with fluorescence detection (Haeuptle et al., 2010). This method, based on a multi-step sample preparation and derivatization procedure, is labor intensive and time consuming. Few mass spectrometry (MS)-based methods exist for the structural characterization of endogenous DolP species (Löw et al., 1991; Wolucka et al., 1996; Guan et al., 2010; Taguchi et al., 2016). To date, a method for the simultaneous profiling and quantification of DolP species of different isoprene chain lengths in biological membranes has been lacking. We have developed a method for the characterization and quantification of DolPs using trimethylsilyldiazomethane (TMSD) derivatization followed by reverse phase (RP) liquid chromatography–high-resolution mass spectrometry (LC–HRMS) analysis. TMSD is frequently used as a methylating agent for anionic phospholipids (Lee et al., 2017) and free fatty acids (Mok et al., 2016) to increase the electrospray ionization efficiency. The scheme of the methylation reaction of DolP is illustrated in Figure 1.

We found that TMSD methylation enabled chromatographic retention of DolPs in RPLC analyses. In the Tandem MS (MS/MS or MS2) analysis of all methylated DolP species by high-energy collisional dissociation, the most intense fragment ion at m/z 127.0155 represents a characteristic headgroup fragment, corresponding to a dimethylphosphate group. Transitions from all parent m/z (Table 1) to 127.0155 in the MS2 spectra were used to determine the identification of methylated DolPs and dodecaprenyl phosphate (PolP C60).

**Table 1.** Molecular formulas, exact masses, and retention times of [M+NH_4_]^+^ of DolP species and the internal standard (IS) PolP C60

Analyte	Molecular formula	Exact mass [M+NH_4_]^+^	Retention time (min)
PolP C60(IS)	C62H103O4P1	960.7932	19.1
DolP C65	C67H113O4P1	1030.8715	20.0
DolP C70	C72H121O4P1	1098.9341	20.6
DolP C75	C77H129O4P1	1166.9967	21.2
DolP C80	C82H137O4P1	1235.0593	21.8
DolP C85	C87H145O4P1	1303.1219	22.3
DolP C90	C92H153O4P1	1371.1845	22.9
DolP C95	C97H161O4P1	1439.2471	23.5
DolP C100	C102H169O4P1	1507.3097	24.1
DolP C105	C107H177O4P1	1575.372	24.7

The established workflow uses a fast and efficient single-step derivatization procedure. Furthermore, the method was used to quantify DolP species from lipid extracts of *Saccharomyces cerevisiae* and HeLa cells. In the future, this method can be used to study the role of DolP homeostasis in health and disease and how DolP availability may be regulated by microenvironmental factors such as nutrient availability. This method has been thoroughly validated for its quantification utility in Kale et al. (2023).

## Materials and reagents

1. Pipette tips, 50–1,000 μL (Eppendorf, catalog number: 613-3505)
2. Gastight syringe, 5 mL (Hamilton, catalog number: 81517)
3. Pipette tips Combitips advanced 25 mL (Eppendorf, catalog number: EP 0030089472)
4. Round bottom threaded glass tubes (PYREX, catalog number: 9447161)
5. Pasteur pipettes, soda-lime glass (Brand, Wertheim, Germany, catalog number: 747715)
6. Micro inserts, Neochrom (Neolab, catalog number: 7-0635)
7. LC vials, Neochrom (Neolab, catalog number: EC-1184)
8. Threaded screw caps with Teflon Liner (Corning, catalog number: 9998-15)
9. Green nitrile gloves (TouchNtuff, catalog number: 92600)
10. Ammonium acetate (AmAc), eluent additive for LC-MS, LiChropur (Sigma-Aldrich, Merck KGaA, catalog number: 73594)
11. Formic acid (FA), eluent additive for LC-MS (Honeywell, catalog number: 56302)
12. Ammonium bicarbonate (Sigma-Aldrich, Merck KGaA, catalog number: 09830)
13. Acetonitrile, LC-MS grade (Fisher Chemical, catalog number: 10616653)
14. Methanol, LC-MS grade (Fisher Chemical, catalog number: 10532213)
15. Isopropanol, LC-MS grade, Optima (Fisher Chemical, catalog number: A461212)
16. Water, LC-MS grade, Optima (Fisher Chemical, catalog number: 10505904)
17. Dichloromethane (Sigma-Aldrich, Merck KGaA, catalog number: 270997)
18. Dolichol 13–21 phosphate mixture (DolP standard hereafter) (Sigma-Aldrich, Merck KGaA, catalog number: 900201X)
19. Dodecaprenyl phosphate (PolP C60) (CymitQuimica, catalog number: 48-62-1060)
20. Mini-BeadBeater q mill beads (0.5 mm) (BioSpec Products, catalog number: 11079105)
21. Potassium hydroxide pellets (KOH) (Sigma-Aldrich, Merck KGaA, catalog number: 21473)
22. Trimethylsilyldiazomethane (TMSD) (Sigma-Aldrich, Merck KGaA, catalog number: 362832)
23. Acetic acid ACS grade (Sigma-Aldrich, Merck KGaA, catalog number: 33209)
24. HeLa/Fibroblast cells (~1 M cells)
25. *S. cerevisiae* cells (~0.8 OD)
26. Acquity UPLC CSH C18 Column, 1.7 μm, 1 mm × 150 mm (Waters, catalog number: 186005294)
27. Acquity UPLC CSH C18 VanGuard Pre-column, 1.7 μm, 2.1 mm × 5 mm (Waters, catalog number: 186005303)
28. For the preparation of liquid chromatography, mobile phase A (Solvent A) and mobile phase B (Solvent B), and wash solvents, please see Recipes.

**Solutions**

1. 15 M KOH (see Recipes)
2. 10 M AmAc (see Recipes)
3. Solvent A: acetonitrile/water 60:40 (v/v) with 0.1 % FA and 10 mM AmAc (see Recipes)
4. Solvent B: isopropanol/water 90:10 (v/v) with 0.1 % FA and 10 mM AmAc (see Recipes)
5. Wash solvent: dichloromethane:methanol:water (3:48:47, v/v/v) (see Recipes)
6. Dichloromethane:methanol mixture (6.5:5.2, v:v) (see Recipes)
7. 155 mM ammonium bicarbonate buffer (see Recipes)

**Recipes**

1. **15 M KOH**
  Accurately weigh 8.4 g of KOH pellets into a 100 mL volumetric flask. Add LC-grade water dropwise with intermittent slow vortex to dissolve KOH. Be careful, as an exothermic reaction will occur, and the reaction flask will be hot. After complete dissolution, dilute the solution to the mark. Use a glass bottle with a PTFE lid for storage of the KOH solution.
2. **10 M AmAc**
  Take the 10 mL volumetric flask along the glass stopper and rinse both twice with MS-grade methanol. Dry them carefully under Argon. Transfer accurately weighed 7.708 g of LC-MS grade AmAc to the volumetric flask. To dissolve the solid, add distilled water in steps of approximately 100 µL, followed by sonication. Once all solids are dissolved, fill the volumetric flask to the etched line. This stock can be stored at 4 °C for up to six months.
3. **Solvent A: acetonitrile/water 60:40 (v/v) with 0.1 % FA and 10 mM AmAc**
  Prepare 1,000 mL of solvent mixture of acetonitrile/water 60:40 (v/v) and add 1 mL of formic acid and 1 mL of 10 mM AmAc stock. Mix solvent mixture thoroughly and degas for 5 min in an ultrasonic water bath sonicator.
4. **Solvent B: isopropanol/water 90:10 (v/v) with 0.1 % FA and 10 mM AmAc**
  Prepare 1,000 mL of solvent mixture of isopropanol/water 90:10 (v/v) and add 1 mL of FA and 1 mL of 10 mM AmAc stock. Mix solvent mixture thoroughly and degas for 5 min in an ultrasonic water bath sonicator.
5. **Wash solvent: dichloromethane:methanol:water (3:48:47, v/v/v)**
6. **Dichloromethane:methanol mixture (6.5:5.2, v:v)**
7. **155 mM ammonium biocarbonate buffer**
  Transfer accurately weighed 1.2 g of ammonium bicarbonate into a 100 mL volumetric flask. Dissolve in distilled water and make up to the mark with distilled water. This stock can be stored at 4 °C for up to six months.

## Equipment

1. Eppendorf Multipette E3 (Eppendorf, catalog number: VB-1748)
2. Eppendorf Research plus pipette 100–1,000 μL (Eppendorf, catalog number: 3123000063)
3. Ultimate 3000 LC system (Dionex, Thermo Fisher Scientific) system coupled to a Q-Exactive HRMS (Thermo Scientific)
4. Vortexer, SI Vortex-Genie 2 (Scientific Industries, catalog number: 15547335)
5. Nitrogen evaporator (TurboVap, catalog number: 416200)
6. Syringe, gastight HamiltonTM1700 Series 100 μL (Sigma-Aldrich, MerckKGaA, catalog number: 111HAM201050)
7. Refrigerated centrifuge, Heraeus Megafuge 16R, (Thermo Scientific, catalog number: 75004271)
8. Ultrasonic water bath sonicator (Bandelin sonorex)
9. Water bath, LSB Aqua Pro (Grant Instruments, catalog number: LSB12)

## Software

1. XCalibur software 3.2 (Thermo Fisher Scientific)
2. Online EnviPat R package Tool (available at https://www.envipat.eawag.ch/, Accessed: 21 August 2019)

## Procedure

**Figure 2.**
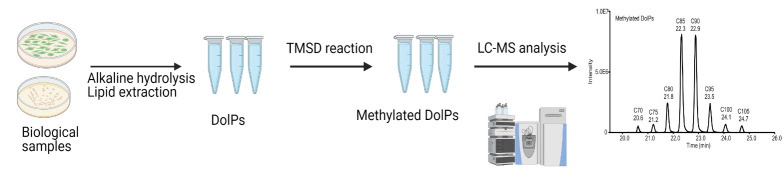
Overview of the developed workflow, including sample preparation, methylation, and reverse phase liquid chromatography–mass spectrometry (RPLC-MS) analysis of dolichyl phosphates (DolPs)

*Note: Below mentioned steps should take place in a cold room or on ice where possible.*

A schematic of the workflow for the analysis of DolPs is shown in Figure 2. The different procedures involved in this workflow are mentioned below.

1. Thaw the biological samples, such as HeLa/fibroblast cells (~1 M cells) or *S. cerevisiae* cells (~0.8 OD), on ice for 20 min.
2. If performing Hela cells extraction, skip this step. Suspend *S. cerevisiae* cell pellets in 200 μL of 155 mM ammonium bicarbonate buffer. Add 0.5 mm glass beads to samples and vortex for 1 min. Then, centrifuge samples at 3,500× *g* for 1 min at room temperature (RT). Transfer the aqueous (top) layer to a clean microcentrifuge tube.
3. Add 20 pmol of internal standard (IS) PolP C60 to cell pellets/lysates.
4. Add 1 mL of methanol and vortex for 1 min at normal speed, followed by sonication for 1 min in an ultrasonic water bath sonicator.
5. Add 1 mL of water and vortex for 1 min at normal speed, followed by sonication for 1 min with an ultrasonic water bath sonicator.
6. Centrifuge at 3,500× *g* for 5 min at RT.

*Note: If you wish to normalize DolP amounts to total lipid/total phosphatidylcholine (PC) (as bulk membrane lipid), measure the total lipid/PC levels of samples at this point. See Özbalci et al. (2013) for the procedure to determine the total lipid/PC levels of samples (Özbalci et al., 2013).*

**Procedure for alkaline hydrolysis and extraction**

*Note: Use round bottom screw-top glass tubes with Teflon-lined caps for DolP sample preparation. Dichloromethane (which is added in subsequent steps) can dissolve plastic materials. Therefore, always use glass bottles, pipettes, and syringes while working with dichloromethane.*

1. Fill the water bath with distilled water to the upper level of 2.5 mL of solvent in a glass tube immersed in the water bath. Heat the water bath to 85 °C.
2. Add 0.5 mL of 15 M KOH to samples to initialize alkaline hydrolysis.
3. Vortex for 1 min, followed by centrifugation at 3,500× *g* for 5 min at RT.
4. Then, immerse glass tubes in a preheated linear shaking water bath at 85 °C for 1 h, under constant shaking at 40 rpm.
*Note: This strong alkaline treatment releases DolP from DolP-linked monosaccharides, dolichyl diphosphate (DolPP)-linked mono- and oligosaccharides, and DolPP (Keller et al., 1985; Lee Adair and Kennedy Keller, 1985; Fernandez et al., 2001; Schenk et al., 2001).*

1. Cool samples to RT. Vortex for 1 min, followed by centrifugation at ~3,500× *g* for 5 min at RT.
2. Increase the level of water in the water bath with distilled water to the upper level of 7.5 mL of solvent in a glass tube immersed in the water bath. Heat the water bath to 40 °C.
3. To the cooled samples, carefully add 1 mL of methanol and 4 mL of dichloromethane to induce phase partitioning.
4. Then, immerse glass tubes in a preheated linear shaking water bath at 40 °C for 1 h, under constant shaking at 40 rpm.
5. Cool the samples at RT; then, vortex and centrifuge at 3,500× *g* for 5 min at RT.
6. Following centrifugation, remove the upper aqueous phase using a Pasteur pipette and wash the lower organic phase four times with wash solvent (see Recipes).
7. Carefully transfer the bottom layer to a new glass tube using a Pasteur pipette.
8. Dry samples under a gentle stream of nitrogen at RT in a Turbovap evaporator.

**Methylation of DolPs**

*Note: Inhaling TMSD may cause lung injury or central nervous system depression. TMSD must be handled with caution and following proper safety standards. Before starting the reaction, ensure that the hood is working properly. When working with TMSD, personal safety equipment such as lab aprons, latex gloves, goggles, and respirators should be worn at all times. Screw cap glass tubes immediately after the addition of the reagents.*

1. QC standard: methylate DolP standard and IS mixture along with samples. In a screw-top glass tube, take 20 pmol of DolP standard and 20 pmol of IS and dry under a gentle stream of nitrogen.
2. Add 200 μL of dichloromethane:methanol mixture (6.5:5.2, v:v, see Recipes) to dried samples.
3. Vortex for 1 min followed by centrifugation at 3,500× *g* for 5 min at RT.
4. To these tubes, carefully add 10 μL of TMSD using a gas-tight Hamilton syringe.
5. Vortex for 1 min, followed by centrifugation at 3,500× *g* for 5 min at RT.
6. Incubate for 40 min at RT.
*Note: The chemical reaction of TMSD (yellow) with the acetic acid produces (colorless) methyl acetate. After adding TMSD to the lipid extracts, the solution should have a faint yellow color. If the yellow color associated with TMSD disappears in the solution, add TMSD in 5 μL increments until a yellow color appears. It is recommended to dry the lipid extracts completely before methylation to avoid neutralization of TMSD.*
7. When the reaction is complete, neutralize excess TMSD by slowly adding 10 μL of acetic acid.
*Note: The color of the solution should change from yellow to colorless after the addition of acetic acid. Add acetic acid in 5 μL increments until the yellow color disappears.*
8. Dry samples under a gentle stream of nitrogen at RT in a Turbovap evaporator.
9. For LC–HRMS analysis, reconstitute dried samples in 120 μL of methanol. Vortex for 1 min and transfer lipid films to micro inserts placed in 1.5 mL Eppendorf tubes.
10. Centrifuge the samples in Eppendorf tubes in a microcentrifuge at 1,500× *g* for 5 min at 4 °C. Transfer micro inserts to LC vials for analysis.

**LC system preparation**

1. Prepare Solvents A and B according to Recipes.
2. Mix each solvent thoroughly by shaking and degas both solvents in the water bath sonicator with loose caps for at least 10 min.
3. Put up both solvents on the LC system and purge all lines for 5 min.
4. Set the flow rate to 0.1 mL/min with both solvents in the ratio 60:40 (solvent A:B).
5. Maintain the column at 55 °C.
6. Equilibrate the column for at least 15 min after the column pressure is stable.
7. Set the LC gradient as follows: 40% B at 0 min, 40%–50% B (0–3 min), 50%–54% B (3–9 min), 54%–70% B (9–9.1 min), 70%–90% B (9.1–17 min), 90% B (17–27.5 min), 90%–40% B (27.5–27.6 min), and 40% B (27.6–30 min).

**MS system preparation**

*Note: Perform mass calibration for the mass spectrometer with XCalibur software.*

1. Set the tune MS parameters to the ESI positive mode, as shown in Table 2.
  **Table 2.** MS parameters used for Q-Exactive Plus high-resolution mass spectrometer

MS parameter	value/unit	MS parameter	value/unit
Spray voltage	3.5 kV	Scan range	960 to 1,600 m/z
Sheath gas	30 L/min	Spectrum data type	Profile
Auxiliary gas	10 L/min	Isolation window	4.0 m/z
Spare gas	1 unit	Collision energy NCE	50%
S-Lens	50 eV	Maximum IT	100 ms
Resolution AGC Target	17,500 at m/z 200 2 × 10^5^ for MS1	Capillary temperature	250 °C
	70,500 at m/z 200 3 × 10^6^ for MS2
2. Inject 100 μL of each sample in the following order:
3. Solvent blank: inject solvent as blank at least three times for gradient stabilization.
4. Then inject QC sample to check the performance of methylation and the LC-MS system.
5. Randomize the sequence of samples.

## Data analysis

**Evaluation of raw data**

1. Raw MS1 and MS2 spectral data were processed, analyzed, and visualized using the XCalibur Qualbrowser as shown in Figure 3.
  **Figure 3.**
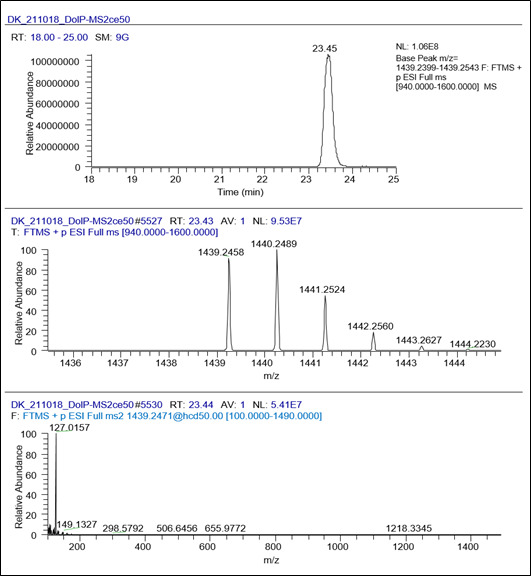
Default layout for the dolichyl phosphates (DolP) C95 example data file using XCalibur Qualbrowser
2. Qualbrowser in XCalibur was used for MS data visualization. The XICs for [M+NH_4_]^+^ ions of commercially available DolP standards and IS were created by extracting theoretical m/z values (shown in Table 1) with a 10 ppm accuracy window.
3. Chromatographic retentions for DolPs and IS were obtained from the most intense peak in XIC traces.
4. Further, structural information of DolP species and IS was derived from MS1, and MS2 obtained at their retention time. A representative XIC, MS1, and MS2 analysis of DolP C95 from DolP standard mixture and HeLa cell extracts is shown in Figure 4.
  **Figure 4.**
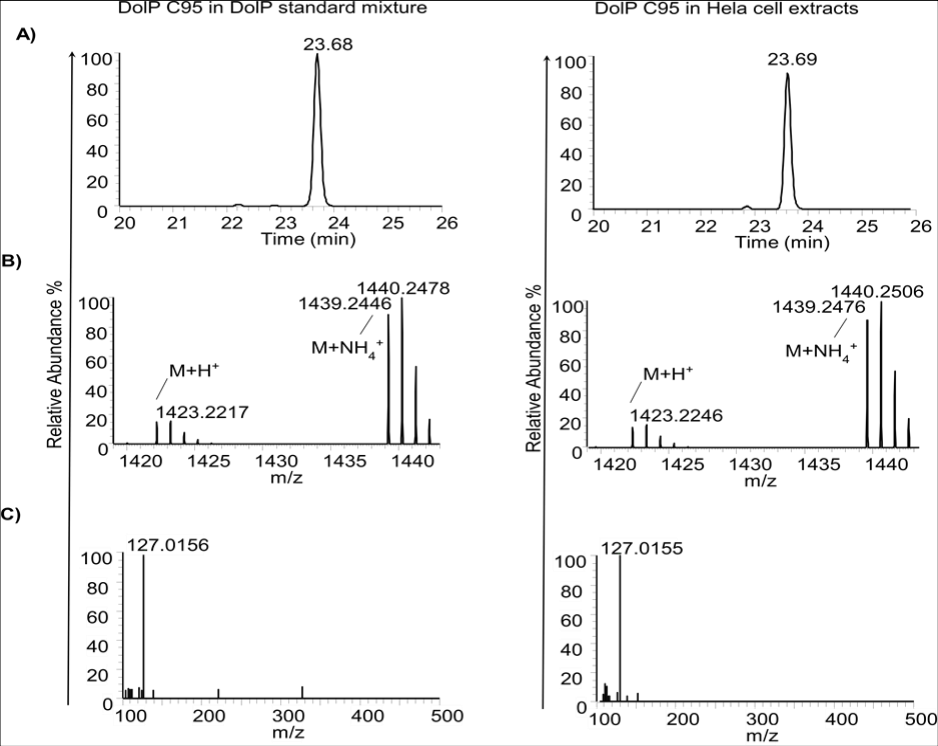
Identification of dolichyl phosphates (DolP) C95 in DolP standard mixture (left panel) and in HeLa cell extracts (right panel) based on A) extracted ion chromatograms (XIC), B) MS spectrum, and C) MS2 spectrum of [M+NH_4_]^+^ ions of DolP C95. For more MS spectral characterization and protocols, please refer to Kale et al. (2023).
5. All methylated DolP species and IS PolP C60 exhibited a characteristic fragment of 127.0155, which is used as a qualifier ion (assigned to the dimethylphosphate group), independent of dolichol or isoprenyl chain length.
6. Endogenous DolP species were identified using matching MS1, retention time, and MS2 spectrum to standards.

**Quantitative analysis of DolP in samples**

1. MS data quantification was established in Quanbrowser by creating a processing method using XCalibur Quanbrowser (Thermo Fisher Scientific XCalibur 3.1 Quanbrowser User Guide, 2014)
2. Get the peak area from the XICs of DolPs and its internal standards based on its accurate m/z value with an accuracy of 10 ppm. The measured peak areas of the methylated DolPs and internal standards must be corrected by multiplying the isotope correction factors. This correction will take into account the differences in the relative 13C natural isotope abundance of different DolP species.
3. Theoretical isotopic distributions were obtained using the online EnviPat R package Tool (Loos et al., 2015). Finally, the concentration of DolPs was estimated by comparing the peak areas of internal standards with known concentrations to the peak areas of DolPs.

## Validation of protocol

The method mentioned in the present protocol has been thoroughly validated according to ICH guidelines [see supplementary information mentioned in Kale et al. (2023)]. The following method validation parameters were tested: lower limit of quantification (LOD) and the linearity range, intra- and inter-day accuracy and precision, extraction recovery, and post-preparative autosampler stability. As described in Kale et al. (2023), the analytical procedure provided acceptable values of validation parameters tested, and the experimental protocol was robust and reproducible.
